# Meta-Analysis of Sex Differences in Social and Communication Function in Children With Autism Spectrum Disorder and Attention-Deficit/Hyperactivity Disorder

**DOI:** 10.3389/fpsyt.2019.00804

**Published:** 2019-11-04

**Authors:** Tania Mahendiran, Jessica Brian, Annie Dupuis, Nadia Muhe, Pui-Ying Wong, Alana Iaboni, Evdokia Anagnostou

**Affiliations:** ^1^Faculty of Medicine, Institute of Medical Science, University of Toronto, Toronto, ON, Canada; ^2^Bloorview Research Institute, Holland Bloorview Kids Rehabilitation Hospital, Toronto, ON, Canada; ^3^Department of Applied Psychology and Human Development, OISE; University of Toronto, Toronto, ON, Canada; ^4^Dalla Lana School of Public Health, University of Toronto, Toronto, ON, Canada; ^5^Map and Data Library, University of Toronto, Toronto, ON, Canada; ^6^Department of Pediatrics, University of Toronto, Toronto, ON, Canada

**Keywords:** autism spectrum disorder, sex differences, attention-deficit/hyperactivity disorder, meta-analysis, neurodevelopmental disorders, social function

## Abstract

**Background:** Sex differences in the prevalence of neurodevelopmental disorders such as autism spectrum disorder (ASD) and attention-deficit/hyperactivity disorder (ADHD) are well documented, but studies examining sex differences in social and communication function remain limited and inconclusive.

**Objectives:** The objective of this study is to conduct a meta-analysis of sex differences in social-communication function in children with ASD or ADHD and typically developing controls.

**Methods:** Using PRISMA, a search was performed on Medline and PSYCHINFO on English-language journals (2000–2017) examining sex differences in social and communication function in ASD and ADHD compared to controls. Inclusion criteria: 1) peer reviewed journal articles, 2) diagnosis of ASD or ADHD and controls, 3) age 6–18 years, 4) measures of social–communication function, and 5) means, standard deviations, and sample sizes reported in order to calculate standardized mean differences (SMD).

**Results:** Eleven original/empirical studies met inclusion criteria for ASD and six for ADHD. No significant sex differences were found between ASD and controls in social (SMD = −0.43; *p* = 0.5; CI: −1.58–0.72), or communication function (SMD = 0.86; *p* = 0.5 CI; −1.57–−3.30) and between ADHD and controls in social function (SMD = −0.68: *p* = 0.7, CI: −4.17–2.81). No studies evaluated sex differences in communication in ADHD. Significant heterogeneity was noted in all analyses. Type of measure may have partially accounted for some variability between studies.

**Conclusions:** The meta-analysis did not detect sex differences in social and communication function in children with ASD and ADHD; however, significant heterogeneity was noted. Future larger studies, controlling for measure and with adequate numbers of female participants are required to further understand sex differences in these domains.

## Introduction

### Rationale

Autism spectrum disorder (ASD) and attention-deficit/hyperactivity disorder (ADHD) are neurodevelopmental disorders, affecting multiple aspects of behavior and cognition (1). Sex differences in prevalence are well documented, but how such sex differences interact/impact core symptom domain phenotypes remains unclear. Given the potential implications for both understanding biology and developing effective interventions, understanding such interactions is critical.

ASD is characterized by deficits in social communication, and repetitive/restricted behaviors, and occurs in approximately 1.5% of children (2, 3). ADHD is characterized by difficulties in attention, hyperactivity, and impulsivity, and has a prevalence of 5–7% in children (4). Comorbidity among these disorders has been reported to be high. The prevalence of comorbid ADHD is reported to be between 30 and 80% in individuals with ASD (5, 6) whereas the presence of ASD is estimated to range between 20 to 50% of individuals with ADHD (7–9). There is also consistent evidence of overlapping behavioral traits, such as inattention, hyperactivity, inhibitory control and other executive functions, repetitive behavior, and social deficits across these disorders, although such symptoms are not always a part of core symptom domains for a specific disorder (6, 10–14).

Both ASD and ADHD are characterized by male predominance. The male to female ratio in ASD has been reported to range from 1.33:1 to 16:1 (15–18). IQ has been reported to influence male to female ratios, with higher ratios (10:1) in individuals with higher IQs and lower ratios (2:1) in individuals with comorbid intellectual disability (15, 19). In ADHD, the male to female ratio is reported to vary between 6:1 (clinical samples) and 3:1 (community samples). Considering the differences in the prevalence of these disorders in males and females, it is important to understand how core symptom presentation may vary by sex.

Social communication deficits are a core symptom of ASD (1), but have also been reported in ADHD. For example, studies have found children with ADHD to have impairments in peer relations and poor friendship quality and stability (20, 21). Some research has argued that social difficulties in children with ADHD may result directly from ADHD symptoms (22, 23) rather than reflecting qualitative impairments in social–communicative function that are characteristic of ASD (24). However, in contrast to this hypothesis, several authors report the presence of social and communicative profiles in ADHD that are qualitatively similar to those associated with ASD (25–27). For example, studies that use the Child Communication Checklist and Social Responsiveness Scale have found that children with ADHD are impaired in a similar manner to many children with ASD (28, 29), suggesting that social–communication impairment in ADHD may not entirely result from ADHD symptoms alone as suggested by Huang-Pollack et al. (22); Tseng and Gau (23). Even though social deficits are seen across these neurodevelopmental disorders, and may indeed have similar presentations, it is unclear how/whether sex differences in prevalence and onset observed in these disorders influence severity of social and communication deficits. Investigating such differences will help us understand the experiences and the unique manifestations/needs of males and females diagnosed with different neurodevelopmental disorders.

There have been relatively few studies in ASD examining sex differences in social–communication function, and findings have been inconsistent. Some studies found that females diagnosed with ASD engaged in significantly more social/peer interaction and had better communication skills compared to males (30–34), while others found no significant differences between males and females (18, 35–37), and some reported that adolescent females had more social–communication difficulties than males (38, 39). Previous systematic reviews have attempted to synthesize inconsistent results and have found no significant differences in social communication function in males and females with ASD. However, these reviews did not include studies with a control group against which to compare findings (40, 41).

Similarly, evidence for sex differences in social–communication function in ADHD remain inconsistent. Most of the literature on ADHD has focused mostly on males and there is limited information on peer relation and social interaction difficulties in females with ADHD (42). While some studies have documented more deficits in peer interaction in males than females (43, 44), other studies found that females were more likely to be rejected/disliked by peers than males (45, 46). Furthermore, a few studies have reported no sex-related differences in social functioning (47–49). To date, the meta-analyses by Gaub and Carlson (50) and Gershon (51) are the only meta-analyses that have examined sex differences in social functioning in children/adolescents with ADHD. Even though both meta-analyses concluded that there were no differences between males and females with ADHD with respect to social/peer functioning, the analyses lacked typically developing control groups, and were performed more than 15 years ago. Thus, some of the study participants were diagnosed with ADHD based on DSM III criteria, but most importantly no studies from the last 15 years were included.

In summary, although sex differences are well documented in the prevalence of neurodevelopmental disorders, and social deficits are observed across such disorders, there is limited research examining how such sex differences may influence social and communication function. Previous attempts at synthesizing available evidence did not include typically developing control (TD) groups, making it unclear whether observed sex differences are similar to those found in the general population or are specific to a condition. Thus, this meta-analysis will attempt to examine whether there are sex differences in social–communication function between children with ASD and ADHD and controls.

### Research Objective

This study will review the current literature in order to examine potential sex differences in social–communication function in children with ASD and ADHD compared to typically developing controls.

## Methods

### Study Design

The current study is a meta-analysis of the literature that will examine sex differences in social and communication function in children diagnosed with ASD and ADHD compared to controls, followed by a meta-analysis of a subgroup of studies to summarize and quantitatively compare sex differences in social and communication function between children with these developmental disorders and controls.

### Search Strategy

A search was performed using OVID Medline and PsychINFO databases for relevant articles in September 2017, on sex differences in social and communication function in ASD and ADHD, using the keywords listed in **Table 1**. Key search terms and Medical Subject Headings terms (MeSH-used for indexing articles) for Medline and PsychINFO for neurodevelopmental disorders, sex differences and social and communication behaviors were selected with the assistance of an academic librarian (PW). During development of key search terms and MeSH headings, the key words, “social” and “communication” were found to produce a more extensive and broader search as these terms captured a wide range of types of social and communication skills, such as social pragmatic skills, verbal and nonverbal communication.

**Table 1:** Key search term and search strings used for the databases OVID Medline and OVID PSYCHINFO.

Category	Search Terms
**Neurodevelopmental Disorders**	1. child development disorders, pervasive/ or Asperger syndrome/ or autism spectrum disorder/ or exp autistic disorder/ 2. exp Child Development Disorders, Pervasive/ 3. Attention Deficit Disorder with Hyperactivity/ 4. autis*.mp. 6. attention deficit.mp. 7. (attention adj3 disorder*).mp. 8. hyperactivit*.mp. 9. All above
**Sex Differences**	10. Sex Factors/ 11. (sex adj3 factor*).mp. 12. (sex adj3 differ*).mp. 13. (male* adj3 female*).mp.14. (boy or boys).mp. 15. (girl or girls).mp. 16. (male* adj3 differ*).mp. 17. (female* adj3 differ*).mp.18. human sex differences/ 19. (gender adj3 differenc*).mp. 20. (gender adj3 profile*).mp.21. sex characteristic*.mp. 22. All above
**Social Behavior and Communication**	23. (social or COMMUNICATION).mp.
**MEDLINE Search Strings including limits**	24. 9 and 22 and 2325. limit 24 to (year = “2000 -Current” and “all child (0 to 18 years)” and English and humans and journal article)
**PSYCHINFO Search Strings including limits**	24. 9 and 22 and 2325. limit 24 to (journal article and english and human and year = “2000–current”)

*represents the truncation symbol for PsychINFO and MEDLINE databases.

### Participant/Comparators

The inclusion criteria were: 1) peer reviewed journal articles, 2) published in English between the year 2000 and 2017, 3) males and females in the sample, 4) diagnosis of ASD or ADHD by DSM criteria and typically developing controls, 4) age range of 6–18 years old, 5) sex differences between the diagnostic group (i.e., ASD or ADHD) and controls tested using measures of social–communication function, and 6) means, standard deviations, and sample sizes reported in order to calculate standardized mean differences (SMD).

### Systematic Review Protocol

Title and abstract of articles were screened for inclusion criteria by two raters (TM, MM). A third rater was consulted in case of discrepancies (EA). In addition, articles were excluded if they were off topic, descriptive studies, did not provide mean scores, standard deviations, and sample sizes for social or communication function for males and females, and/or did not include a typically developing control group. Authors of excluded articles were contacted to request data on control groups for the inclusion in the analysis, but none provide the requested information.

### Data Extraction

We used the Quality Assessment Tool for Cohort and Cross-Sectional Studies to assess the quality of the studies (please see **Supplemental Tables 1 and 2**). Variables extracted for analysis included mean age and standard deviation, sample sizes of males and females with a developmental disorder and controls, type of measure used to assess social and/or communication function, mean scores and standard deviations for females and males on these measures.

### Data Analysis

Random-effects meta-analyses were performed using the “metafor” package in R (52, 53; R Project for Statistical Computing, RRID: SCR_001905) for measures of social and communication function in ASD and social function in ADHD. We used a random-effects model to account for variance within and between studies caused by sampling error and other artefacts (54). Standardized mean sex differences for social and communication function were calculated in ASD and for social function in ADHD. We then calculated SMD between groups. Social and communication function were analyzed separately since some studies tested these individually or only tested for one of these. Where tests for heterogeneity were significant, a mixed-effects model was used to test for the effect of the moderator “Measure” (test/instrument used), as well as “Age” [age of participant-categorized into child (6–12 years); child/adolescent (for studies including both children and adolescents); adolescent (12–18 years)]. ASD groups were entered into the analysis first. Positive effect sizes represent females outperforming males more the in ASD relative to controls, while negative effect sizes represent males outperforming females more in ASD relative to controls. Where multiple measures of the same symptoms were used within one study, we report on measures that were commonly used in other studies. A few studies had more than one measure that assessed social and/or communication behaviors. For the ADHD articles, some articles used more than one measure to assess social function (55–57). To maintain consistency across all studies, measures were selected if they assessed social behavior and if they were parent reports (i.e. My Child—55; Social Adjustment Inventory for Children and Adolescents—56; Quality of Play Questionnaire—57). For ASD social function, we found that three studies had reported both the total scores and social communication domain scores of the SRS (58–60). To determine whether we should report the effect size of the total score versus the social communication domain score, the effect sizes of the SRS total scores and social communication domain scores were plotted on to a forest plot and were compared. As both were found to have similar effect sizes and to stay consistent with studies that publish total scores, we decided to use the SRS total scores. Additionally, given that two of the ADHD studies (56 and 61) used community rather than clinic samples, we ran the analyses with and without them. All R scripts for all analyses were borrowed from Dr. Laura Hull (40) and slightly modified with her permission. The R-Script used in the present study is available upon request.

## Results

### Study Selection and Characteristics

**Figure 1:**
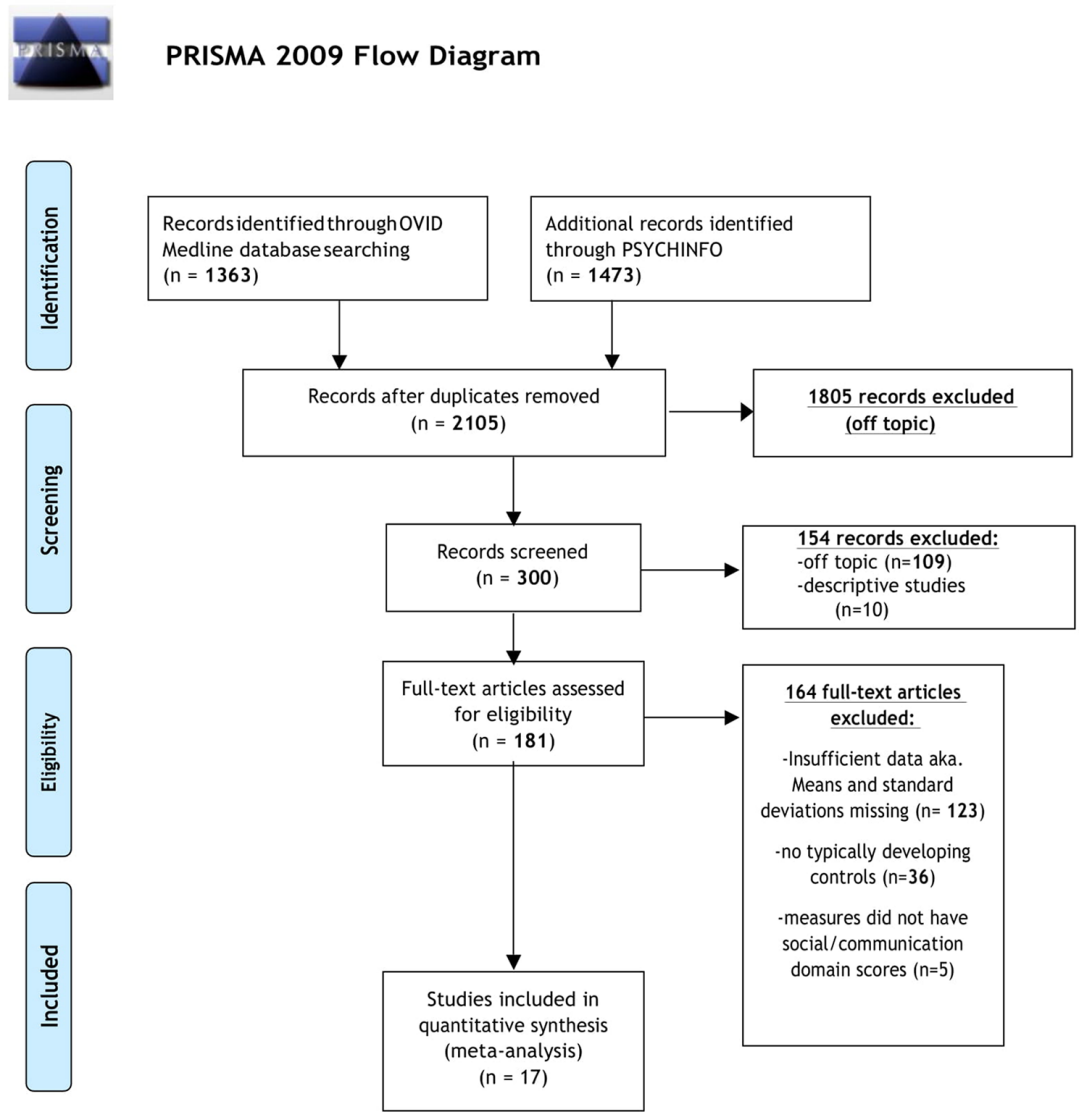
PRISMA flow diagram displaying article selection process. Flow chart from: (62).

The initial database search identified 2,105 results (**Figure 1**). Of the 2,105 studies found, 1,805 were excluded based on title review, which led to 300 articles available for abstract review. From those, articles were excluded if they were off topic (*n* = 109) or were descriptive studies (*n* = 10). Of the remaining 181 articles, 164 articles were excluded after a thorough examination of the data provided (123 articles did not provide mean scores, standard deviations, and sample sizes for social or communication function/deficit for males and females, 36 articles did not include a typically developing control group while 5 articles did not report social–communication scores on any measures). Only 17 original/empirical studies met the inclusion criteria; 11 studies measuring social–communication function in ASD and 6 studies measuring social function in ADHD. **Figure 1** provides a detailed overview of this selection process. A summary of the quality of the studies included is seen in **Supplemental Tables 1 and 2**. All studies were cross sectional in nature. All but two studies represented clinical samples, which are associated with high risk of bias. Study demographics for ASD and ADHD are presented in and **Tables 2** and **3** respectively.

**Table 2:** Autism spectrum disorder (ASD) demographic information.

Author				ASD							
	IQ Measure Used	IQ*	Age Range	Mean Age (SD)	Female (n)	Male (n)	Total (n)	Mean Age (SD)	Female (n)	Male (n)	Total (n)
Cholemkery et al. (59)	–Hamburg–Wechsler Intelligence Test for children–WIE or the CFT 20-R for adults	-ASD: 102.15 (SD 16.23),-TD:105.32 (SD 11.62)	6–18 Child/adolescent	12.28 (3.03)	17	43	60	11.18 (3.32)	18	24	42
Cholemkery et al. (60)	–Hamburg–Wechsler Intelligence Test for children–WIE or the CFT 20-R for adults	–ASD: 100.6 (SD 15.2)–TD group is 103.4 (SD 14.5)	6–18 Child/adolescent	12.5 (2.7)	8	47	55	11.9 (2.9)	10	45	55
Head et al. (32)	Not reported	70 or above	10–16 Child/adolescent	13.73(1.97)	25	25	50	12.00 (1.84)	25	26	51
Horiuchi et al (63)	WISC-III or WISC-IV	–Full IQ: 88.3 (20.1), range: 40–132–28 had an intellectual disability	4–16 Child/adolescent	7.92 (3.28)	44	129	173	7.92 (3.28)	44	129	173
May et al. (58)	WISC-IV or WASI	70 or above	7–12 Child	12.96 (1.11 )	32	32	64	12.67( 0.89)	30	30	60
Park et al. (64)	Korean version of the Leiter International Performance Scale	50 or above–No significant sex difference in ASD (*p* = 0.8) and TD (*p* = 0.4)	4–15 Child/adolescent	M: 8.36 (2.79) F: 8.17 (3.37)	20	91	111	M: 8.94 (1.59) F: 8.31 (2.21)	25	26	98
Sedgewick et al. (34)	WASI	Not reported	12–16 Adolescent	M:13.10(1.0)F:13.6(1.1)	13	10	23	M:14.0(1.1)F:14.0(0.11)	13	10	23
Solomon et al. (37)	WASI	–Range from 76 to 145 in ASD and 98–139 in TD–No significant sex difference (did not report stats)	8–18 Child/adolescent	M:12.45(3.72)F:12.0(3.42)	20	20	40	M:12.53(3.32)F:11.42(2.37)	19	17	36

ASD, autism spectrum disorder; TD, typically developing controls; SD, standard deviation; M, males; F, females; WIE, Wechsler Intelligence Test; CFT 20-R, revised Culture Fair Intelligence Test; WISC, Wechsler Intelligence Scales for Children; WASI, Wechsler Abbreviated Scales of Intelligence.

*IQ information is limited to what was reported in the studies.

**Table 3:** ADHD demographic information.

Author	IQ Measure	IQ		ADHD							
			Age Range	Mean Age (SD)	Female (n)	Male (n)	Total (n)	Mean Age (SD)	Female (n)	Male (n)	Total (n)
Biederman et al. (56)	Wechsler intelligence test–Full scale IQ	–80 or greater	6–17 Child/adolescent	M:12.6(4.7)F: 13.6(4.4)	25	73	98	M:13.4(5.5)F:13.7(5.5)	235	244	479
Graetz et al. (61)	Not reported	Not reported	6–13Child/adolescent	M:9.2(2.4)F:8.9(2.4)	26	76	102	M:9.6(2.3)F:9.5(2.3)	1,075	976	2,051
Marton et al. (55)	WISC-IV or Wechsler Intelligence Scale for Children	–80 or greater–ADHD 103.6 (SD = 12.8)–TD was 112.0 (SD = 12.5)	8–12 Child	10.08 (1.39)	14	36	50	10.20 (1.46)	12	30	42
Skogli et al. (65)	WASI–Full scale IQ	–70 or greater–Female controls were significantly higher than males and females with ADHD [*F*(3,126) = 4.6.*p* = 0.004)	8–17 Child/adolescent	11.2	37	43	80	11.9	18	32	50
Rucklidge and Tannock, (66)	Wechsler Intelligence Scale for Children-Full Scale IQ	–80 or greater	13–16 Adolescent	M:14.80(1.22)F:14.68(1.51)	24	35	59	M:14.80(1.22)F: 15.60(1.04)	28	20	48
Mikami and Lorenzi (57)	Wechsler Intelligence Scale for Children-fourth edition	–Verbal IQ 75 or greater–Verbal IQ between ADHD and TD groups were significantly significant *F*(1,121) = 18.94, *p* < 0.01)	6–10 Child	M:8.24(1.14)F:8.19(1.44)	21	42	63	M:8.33(1.28)F:8.10(1.07)	20	42	62

ADHD, attention-deficit/hyperactivity disorder; TD, typically developing controls; SD, standard deviation; M, males; F, females; WISC-IV, Wechsler Intelligence Scales for Children–Fourth Edition ; WASI, Wechsler Abbreviated Scales of Intelligence.

*IQ information is limited to what was reported in the studies.

**Figure 2:**
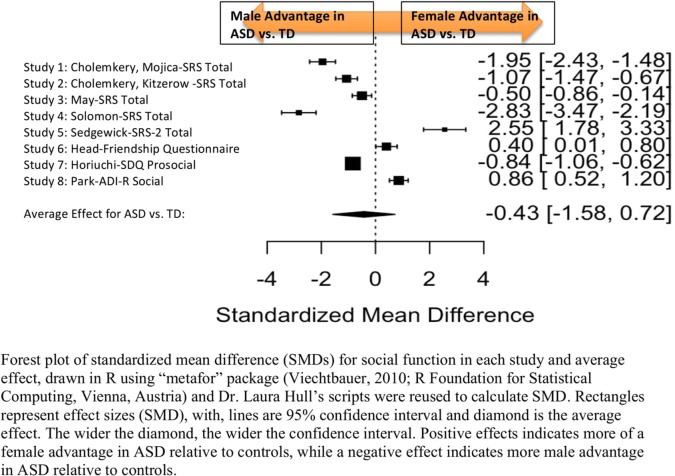
Meta-analysis of studies comparing sex differences in social abilities between ASD and controls. Forest plot of standardized mean difference (SMDs) for social abilities in each study and average effect, drawn in R using “metafor” package (48; R Foundation for Statistical Computing, Vienna, Austria) and Dr. Laura Hull’s scripts were reused to calculate SMD. Rectangles represent effect sizes (SMD), with, lines are 95% confidence interval and diamond is the average effect. The wider the diamond, the wider the confidence interval. Positive effects indicates more of a female advantage in ASD relative to controls, while a negative effect indicates more male advantage in ASD relative to controls.

**Figure 3:**
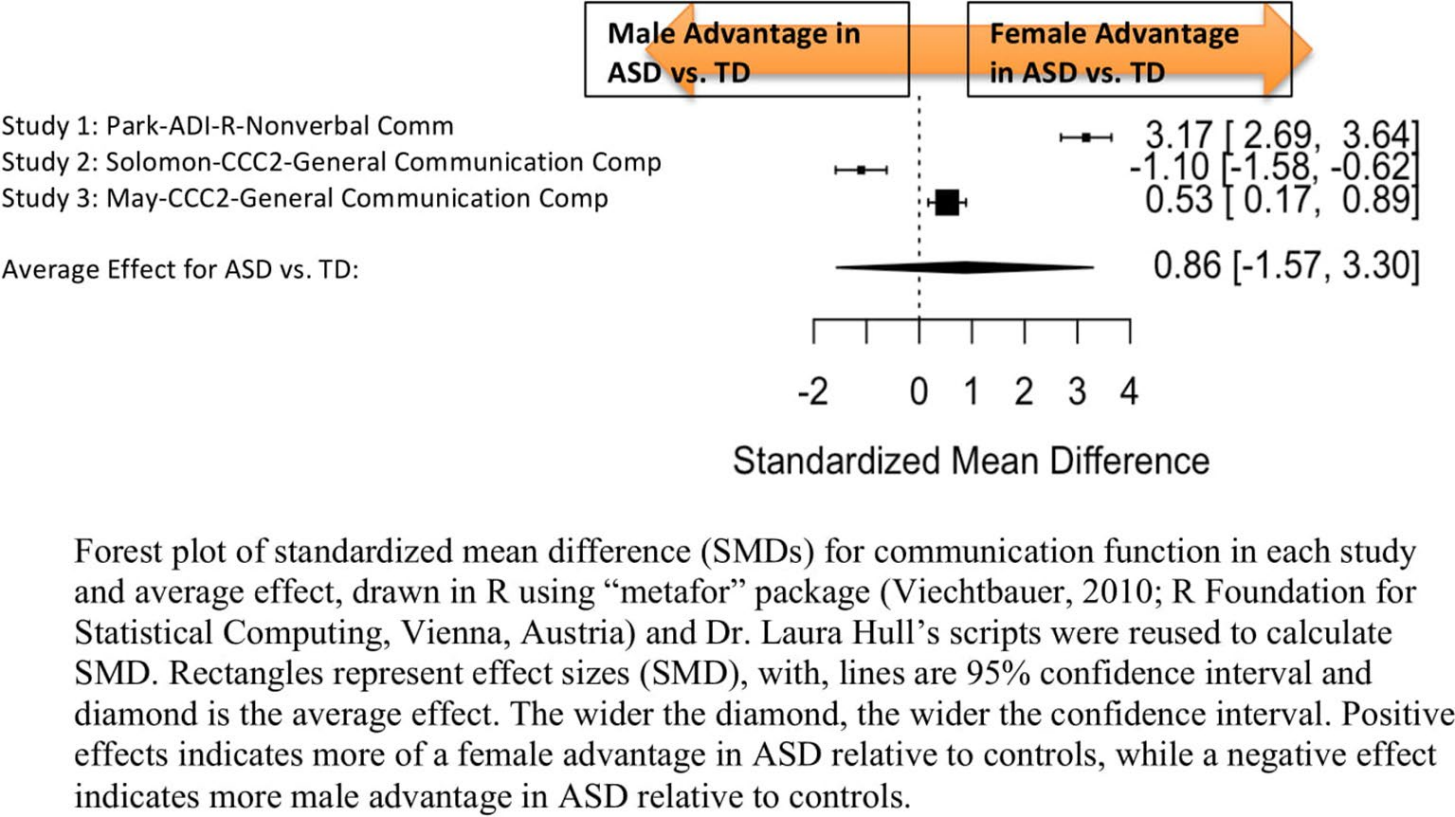
Meta-analysis of studies comparing sex differences in communication abilities between autism spectrum disorder (ASD) and controls. Forest plot of standardized mean difference (SMDs) for communication abilities in each study and average effect, drawn in R using “metafor” package (48; R Foundation for Statistical Computing, Vienna, Austria) and Dr. Laura Hull’s scripts were reused to calculate SMD. Rectangles represent effect sizes (SMD), with, lines are 95% confidence interval and diamond is the average effect. The wider the diamond, the wider the confidence interval. Positive effects indicates more of a female advantage in ASD relative to controls, while a negative effect indicates more male advantage in ASD relative to controls.

**Figure 4:**
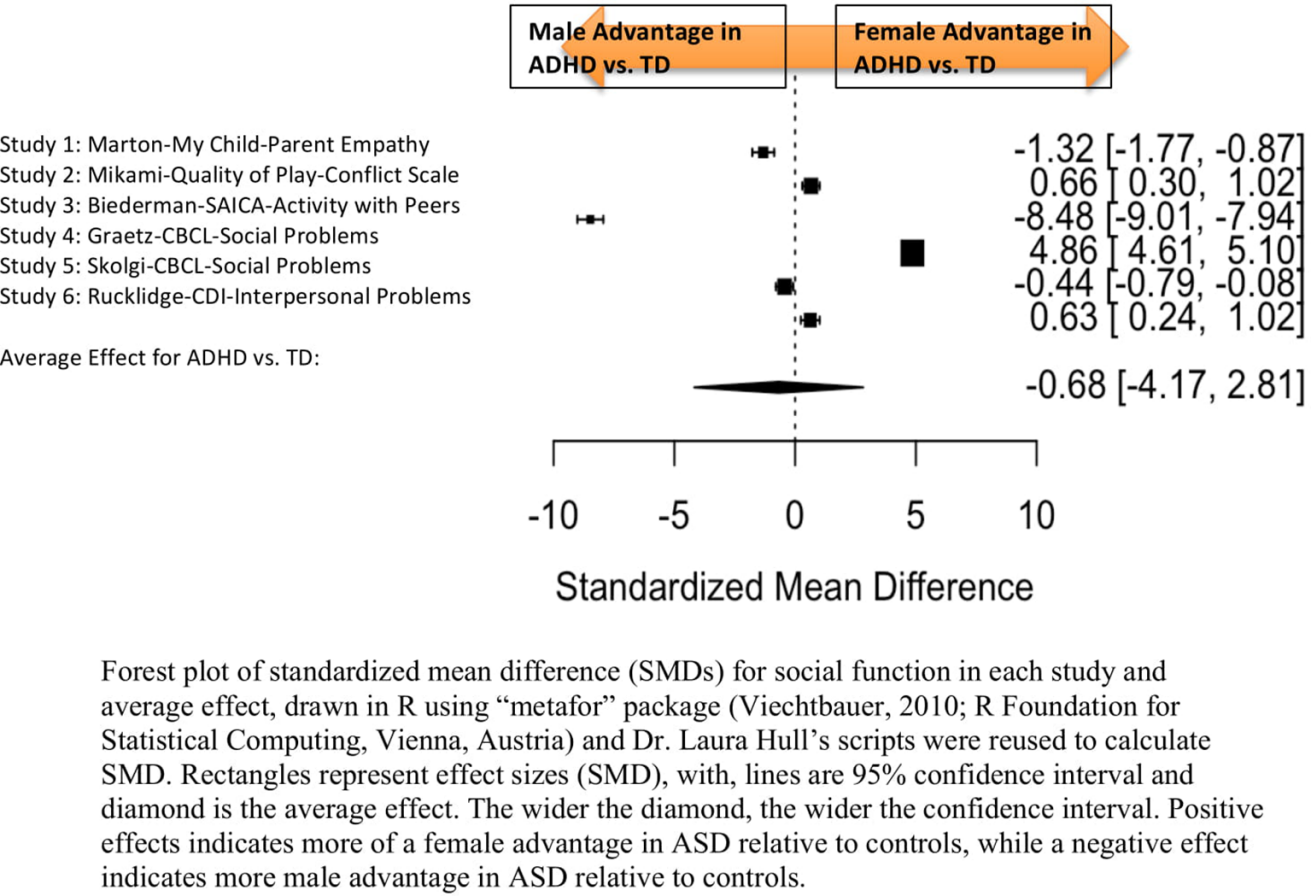
Meta-analysis of studies comparing sex differences in social abilities between attention-deficit/hyperactivity disorder (ADHD) and controls. Forest plot of standardized mean difference (SMDs) for social abilities in each study and average effect, drawn in R using “metafor” package (48; R Foundation for Statistical Computing, Vienna, Austria) and Dr. Laura Hull’s scripts were reused to calculate SMD. Rectangles represent effect sizes (SMD), with, lines are 95% confidence interval and diamond is the average effect. The wider the diamond, the wider the confidence interval. Positive effects indicates more of a female advantage in autism spectrum disorder (ASD) relative to controls, while a negative effect indicates more male advantage in ASD relative to controls.

Standardized mean sex differences for social and communication function were calculated in ASD (**Tables 4** and **5**), and for social function in ADHD (**Table 6**). SMD were then computed between groups using the “metafor” package in R software (52, 53; R Project for Statistical Computing, RRID: SCR_001905), to yield pooled SMDs; the pooled SMDs are represented in the forest plots in **Figures 2**–**4**. Please note that since higher scores represent more impairment in some measures but better abilities in others, signs on scores were changed to ensure higher scores indicate less impairment on all measures. A positive effect size indicates that females outperformed males.

**Table 4:** Sex differences in social function for ASD and TD.

Authors	Social Measures	Community vs. Clinic samples	Age	ASD				TD	
Social				Female (SD)	Male (SD)	SMD (95% CI)	Female (SD)	Male (SD)	SMD (95% CI)
Cholemkery, (59)	SRS Total	Clinic	6–18Child/adolescent	−113.00(24.2)*	−92.95(24.98)*	−0.80(−1.40, −0.20)	−22.94(12.75)*	−20.33(12.54)*	−0.20(−0.80,0.41)
Cholemkery, (60)	SRS Total	Clinic	6–18Child/adolescent	−111.90 (25.70)*	-94.50 (26.30)*	−0.65(−1.40,0.10)	−22.20(15.40)*	−18.8(12.50)*	−0.26(−0.90,0.40)
Head et al. (32)	The Friendship Questionnaire	Clinic	10–16Child/adolescent	76.76 (13.97)	61.48 (15.64)	1.01(0.43,1.60)	84.84 (9.91)	74.76 (12.15)	0.89(0.32,1.47)
Horiuchi et al. (63)	SDQ-Prosocial	Clinic	4–16Child/adolescent	4.30(2.80)	4.28(2.50)	0.01(−0.33,0.35)	6.02(2.00)	5.71(2.00)	0.15(−0.19,0.50)
May et al. (58)	SRS Total	Clinic	7–12Child	−97.41(31.77)*	−99.97(22.71)*	0.09(−0.40,0.58)	−23.17(16.49)*	−27.30(20.42)*	0.22(−0.29,0.73)
Park et al. (64)	ADI-R Social Subscale	Clinic	4–15Child/adolescent	−8.55 (4.43)*	−10.25 (3.83)*	0.43(−0.06,0.92)	−1.00(1.22)*	−1.28 (1.46)*	0.20(−0.35,0.75)
Sedgewick et al. (34)	SRS-2 Total	Clinic	12–16Adolescent	−72.00(32.39)*	−103(27.76)*	0.98(0.11,1.85)	−43(13.18)*	−40.00(26.16)*	−0.15(−0.97,0.68)
Solomon et al. (37)	SRS Total	Clinic	8–18Child/Adolescent	−103.85(27.64)*	−104.60(32.04)*	0.02(−0.60,0.64)	−18.11(18.79)*	−62.12(60.81)*	0.98(0.29,1.67)

Table displays, measures that assess social abilities, age, mean scores, and standard deviations for females and males, and calculated standardized mean differences between females and males in autism and typically developing controls.

ASD: autism spectrum disorder; TD, typically developing controls; SD, standard deviation; SMD, standardized mean difference; CI, confidence interval; SRS, Social Responsiveness Scale; SDQ, Strengths and Difficulties Questionnaire; ADI, Autism Diagnostic Interview–Revised

*Please note, that since higher scores represents more impairment in some measures, while other measures had higher scores mean less impairments, to maintain consistency among the measures, signs on the male and female mean scores were changed to ensure higher scores means less impairment for all measures.

**Table 5:** Sex differences in communication function for ASD and TD.

Authors	Communication Measures	Community vs. Clinic	Age	Autism				TD	
				Female (SD)	Male (SD)	SMD (95% CI)	Female (SD)	Male (SD)	SMD (95% CI)
May et al. (58)	Children’s Communication Checklist (2^nd^ Edition)–General Communication Composite	Clinic	7–12 Child	36.75 (15.05)	33.19 (16.00)	0.23(−0.27,0.70)	80.60 (22.94)	78.63 (19.78)	0.09(−0.42,0.60)
Park et al. (64)	ADI-R nonverbal communication subscale	Clinic	4–15 Child/adolescent	−17.75 (8.20)*	−22.31(6.16)*	0.69(0.20,1.18)	−1.80 (2.33)*	−1.50 (1.90)*	−0.14(−0.70,0.40)
Solomon et al. (37)	Children’s Communication Checklist (2^nd^ Edition)–General Communication Composite	Clinic	8–18 Child/adolescent	76.00 (14.93)	80.95 (24.55)	−0.24(−0.90,0.40)	113.05 (16.20)	111.00(16.37)	0.12(−0.53,0.80)

Table displays, measures that assess communication abilities, age, mean scores, and standard deviations for females and males, and calculated standardized mean differences between females and males in autism and typically developing controls.

ASD, autism spectrum disorder; TD, typically developing controls; SD, standard deviation; SMD, standardized mean difference; CI, confidence interval.

* Please note, that since higher scores represents more impairment in some measures, while other measures had higher scores mean less impairments, to maintain consistency among the measures, signs on the male and female mean scores were changed to ensure higher scores means less impairment for all measures.

**Table 6:** Sex differences in social function for ADHD and TD.

Authors	Social Measures	Community vs. Clinic	Age	ADHD				TD	
Social				Female (SD)	Male (SD)	SMD (95%CI)	Female (SD)	Male (SD)	SMD(95% CI)
Biederman et al. (56)	Social Adjustment Inventory for Children and Adolescents score–Activity with peers	Community	6–17 Child/adolescent	−2.70 (0.60)*	−2.10 (0.80)*	−0.79(−1.30,−0.30)	−1.60 (0.60)*	−1.80 (0.70)*	0.31(0.10,0.50)
Graetz et al. (61)	Child Behaviour Checklist-Teacher’s Report Form–Social problem	Community	6–13 Child/adolescent	−4.00 (3.10)*	−4.80 (3.10)*	0.26(−0.20,0.70)	−1.20 (1.60)*	−1.10 (1.60)*	−0.06(−0.20,0.00)
Marton et al. (55)	Index of Empathy for Children andAdolescents–Child Empathy	Clinic	8–12 Child	72 (10.6)	68.49 (8.97)	0.37(−0.30,1.00)	78.58 (5.24)	73.23 (6.89)	0.81(0.12,1.50)
Skogli et al. (65)	Child Behaviour Checklist–Social Problems	Clinic	8–17 Child/adolescent	−60.00 (7.40)*	−60.40 (9.20)*	0.05(−0.40,0.50)	−50.30 (0.50)*	−50.50 (1.50)*	0.16(−0.40,0.70)
Rucklidge et al. (75)	Children’s Depression Inventory–Interpersonal Problems	Clinic	13–16 Adolescent	−54.67(12.10)*	−50.76(10.84)*	−0.34(−0.90,0.20)	−48.68 (10.01)*	−44.55 (2.70)*	−0.52(−1.10,0.10)
Mikami et al. (57)	Quality of Play Questionnaire-Conflict Scale	Clinic	6–10 Child	−0.91(0.81)*	−0.69(0.70)*	−0.04(−0.40,0.30)	−0.19(0.32)*	−0.16(0.20)*	0.12(−0.40,0.70)

Table displays, measures that assess social abilities, age, mean scores, and standard deviations for females and males, and calculated standardized mean differences between females and males in ADHD and typically developing controls.

ADHD, attention-deficit/hyperactivity disorder; TD, typically developing controls; SD, standard deviation; SMD, standardized mean difference; CI, confidence interval.

*Please note, that since higher scores represents more impairment in some measures, while other measures had higher scores mean less impairments, to maintain consistency among the measures, signs on the male and female mean scores were changed to ensure higher scores means less impairment for all measures.

### Synthesized Findings

#### ASD Social Domain

##### Main Effects

**Table 4** displays the measures used to assess social function, male and female individual mean scores, and the calculated SMD between males and females in ASD and TD groups. No significant sex differences in social function in ASD compared to TD were found (**Figure 2**) (SMD = −0.43, *p*-value = 0.5). Of note, no significant sex differences were noted in social function within ASD (Online Resource 1, **Supplemental Figure 1**) (SMD = 0.13, *p* = 0.6) or within TD (Online Resource 1, **Supplemental Figure 1**) (SMD = 0.24, *p* = 0.1) either. Significant heterogeneity was found in this analysis [*Q*(*df* = 9) = 345.45, *p* < 0.0001], therefore, measure and age were included in the model.

##### Effect of Measure

Measure was not significant in the random effect model [QM(*df* = 5) = 0.14, *p* = 0.7].

##### Effect of Age

Age was also found not to be significant [QM(*df* = 3) = 5.88, *p* = 0.1].

#### ASD Communication Domain

##### Main Effects

**Table 5** displays the measures used to assess communication function, male and female individual mean scores, and the calculated SMD between males and females in ASD and TD groups. A random-effects meta-analysis revealed no significant sex differences between ASD and TD (**Figure 3**) (SMD = 0.86, *p* = 0.5). Of note, no significant sex differences were found within ASD (SMD = 0.25, *p* = 0.3) or TD (Online Resource 1, **Supplemental Figure 2**) (SMD = 0.019, *p* = 0.9) either. Significant heterogeneity was found in this analysis [*Q*(*df* = 2) = 155.66, *p* < 0.0001], therefore, moderators of measure and age were individually evaluated.

##### Effect of Measure

Measure was found to be significant in the random effect model [QM(*df* = 2) = 7.58, *p* = 0.02]. The resulting mixed-effects meta-analysis found significant variation in sex differences for communication function between ASD and TD groups only for the Autism Diagnostic Interview-Revised (ADI-R)–Nonverbal Communication (*p* = 0.006) in one study. However, the test for residual heterogeneity after including “measure” as a moderator remained significant [QE(*df* = 1) = 28.23, *p* < 0.0001], suggesting that other moderators may still be at play.

##### Effect of Age

Age was found not to be significant in the model [QM(*df* = 2) = 0.27, *p* = 0.9].

#### ADHD Social Domain

##### Main Effects

**Table 6** displays the measures used to assess social function, male and female individual mean scores, and the calculated SMD between males and females in ADHD and TD groups. A random-effects meta-analysis revealed no significant sex difference in social function between ADHD or TD (**Figure 4**) (SMD = −0.68, *p* = 0.70). Of note, there were no significant sex differences in social function within ADHD (SMD = −0.038, *p* = 0.84) and TD (Online Resource 1, **Supplemental Figure 3**) (SMD = 0.11, *p* = 0.42) either. Significant heterogeneity was found in this analysis [*Q*(*df* = 5) = 2,316.76, *p* < 0.0001], therefore, moderators of measure and age were included in the model.

##### Effect of Measure

Measure was found to be significant [QM(*df* = 5) = 5.48, *p* = 0.019]. The resulting mixed-effects meta-analysis found a significant variation in sex differences for social function between ADHD and TD groups using the Social Adjustment Inventory for Children and Adolescents–Activity with peers (*p* = 0.024) in one study but not for the rest of the measures (*Child Behavior Checklist*–Social Problems (*n* = 2), Children’s Depression Inventory–Interpersonal Problems (*n* = 1), My Child–Parent Empathy (*n* = 1), Quality of Play–Conflict Scale (*n* = 1)]. Still, the test for residual heterogeneity was significant [QE(*df* = 1) = 571.57, *p* < 0.0001] indicating that other moderators, not included in the model, may still be influencing the effect.

##### Effect of Age

Age was not found to be a significant moderator [QM(*df* = 3) = 0.19, *p* = 0.98].

##### Community Versus Clinic Sample

Graetz (61) and Biederman (56) were the only studies that used community samples instead of clinic samples. When the meta-analysis was conducted excluding the community samples, no significant sex differences emerged [SMD: –0.113; *p* = 0.8106; confidence interval (−1.04–0.81)].

## Discussion

### Summary of Findings

This study examined potential sex differences in social and communication function in neurodevelopmental disorders (i.e., ASD and ADHD) and typically developing groups. The meta-analysis found no evidence of sex differences between ASD and TD groups in social or communication function. Still, with only three studies examining sex differences in communication between ASD and TD, the strength of evidence remains limited. There were no studies examining sex differences in communication function for ADHD. We found no sex differences between ADHD and TD groups in social function. However, the type of measure may partly explain some of the heterogeneity across studies in the domain of communication in ASD and social in ADHD, although only a single study in each disorder was found to be a significant source of heterogeneity and as such other unreported characteristics of these studies such as population characteristics and social economic status may have been responsible for the finding. In summary, there were no sex differences found in social–communication function between ASD and TD and ADHD and TD. However, the choice of measure across studies may have influenced results in some domains but this effect was only seen in one study in each case. Also, given there was significant residual heterogeneity, the variability between studies could have been caused by other factors (e.g. socio-economic status, population characteristics).

Several biological theories have attempted to describe/explain sex differences in developmental disorders. According to Eme (67), the sex least frequently affected by the developmental disorder (females) is relatively more severely affected. Eme (67) explained this using two types of models 1) the polygenetic multiple-threshold model, which suggests that females require a higher genetic/environmental load to be affected, 2) constitutional variability model, which proposes that greater genetic variability in males produces higher rates of less severe manifestations of disorders, while females are more likely to be affected in cases where there is a pathological event (e.g. brain damage). This theory is also consistent with other models used to explain sex differences in ASD such as the Genetic Variability Model (68) and Liability Threshold Model (69). The extreme male brain theory (70) suggests that both males and females with ASD present with an “extreme male” profile of good systematizing abilities at the expense of empathizing abilities, so that fewer sex differences in social communication may be predicted (30, 70). Our findings would partially support the extreme male brain theory, as we found no differences between ASD males and females, although we also did not find sex differences in social function and communication in controls. The latter, although consistent with previous systematic reviews in typically development (70, 72), would not be consistent with the extreme male brain theory. Still several limitations of the identified studies preclude strong conclusions.

To explain potential sex differences in ASD, a few social theories have articulated possible scenarios. Holtmann (38) developed a term called the “interpreting bias” which is the difference between observed and expected behaviors. Holtman (38) suggested that despite comparable levels of ASD traits in males and females on direct measures, parents with children with ASD may expect more socially sophisticated behaviors in their daughters than in their sons, and hence will report more social impairment in their girls than in their boys. Similarly, Crick and Zahn-Waxler (73) reported that girls with ASD were perceived by parents as having a greater level of social impairment, despite comparable symptoms reported and directly observed on the ADI-R and Autism Diagnostic Observation Schedule. Another possible explanation about sex differences in ASD is the increase in social demand/complexity with age may differ between boys and girls. McLennan (39) found more impairment with age in girls but not boys, and suggested that as the child transitions into adolescence, social situations may get more diverse and complex for females, as peer activities in typical girls and young women become mostly dependent on communication and interpersonal skills compared to boys who may have other social options that are less verbal and less intensely interactive (e.g. spectator sports and competitive play). Thus, social deficits may become more evident in girls as they transition to adolescence compared to boys. Another key social factor that has been reported to influence sex differences relates to gender specific expectations related to play and social roles. Despite similar amounts of socializing, Kuo et al. (74) found that males with ASD tended to play video games, whereas females with ASD mostly talked with their friends, suggesting that these skills may allow females with ASD to maintain closer and more empathetic friendships, ultimately to interact as expected by their nonautistic female peers. However, our study results cannot at this point inform these theories as we found no consistent sex differences.

In addition, significant heterogeneity was observed among studies. There are several reasons why there may be discrepancies among studies examining sex effects in ASD and ADHD:

1. Measurement issues: Variability in measures that may be capturing unique constructs, or have differences in psychometric properties. For example, Marton et al. (55) used the parent reported measure “My Child” which assesses only empathic ability, while Skolgi et al. (65) and Graetz et al. (61), used the Child Behavior Checklist Social Problems domain which surveys a broader range of social problems. In the ASD communication domain, the ADI-R assesses social and communication symptoms relevant to ASD while the Children’s Communication Checklist-2 assesses communication skills such as language structure, pragmatic skills and communication skills that are not diagnostic specific.
2. Population differences: Most studies included clinical samples, which may be subject to referral and identification bias. Studies of clinical samples may include more severe cases and/or symptoms that draw more attention, potentially influencing the expression of ASD and ADHD in males and females in the results (76). In fact, a meta-analysis by Gaub and Carlson (50) found that clinic referred females significantly differed from nonreferred females with ADHD, such that clinic-referred females exhibited more severe symptoms and disruptive behaviors. Moreover, girls are more likely to have inattentive symptoms/subtype (77), which may go less noticed and be less likely to lead to a referral and/or ADHD diagnosis compared to the other subtypes. Even though the present study used a random effects model to account for such variances, most studies in this meta-analysis are from clinic populations, and so it is possible that sex differences were examined in children who had more disruptive and severe symptoms.

### Limitations

There were certain limitations in this study. A key limitation was the small number of studies identified. According to Hunter and Schmidt (54), a meta-analysis based on a small number of studies is more susceptible to second-order sampling errors, which may inflate the observed variance. Moreover, several of the studies had very few females included, and may be underpowered to detect sex differences. Further, the choice of measure was identified to be a potential confounding variable, albeit only in single studies, but the residual heterogeneity remained significant, indicating that there were other confounding variables that were influencing sex differences. Previous studies have implicated IQ, ethnicity, and comorbidities (18), as well as other social and biological factors, (genetic influences, social/cultural environments; 76, 78) in interacting with potential sex differences but we had no access to such data. Also, since most of these studies used parent reported measures, results may have been influenced by parental expectations or biases (i.e., the “interpreting bias” described by 38). Moreover, there was some variability in the types of constructs evaluated by measures used in the meta-analysis; while the majority of measures evaluated deficits, other measures may have measured skills. However, there was no evidence that in this set of studies, sex differences were different across the two constructs.

Future research investigating sex differences across neurodevelopmental disorders should include large cohorts with adequate numbers of female participants with neurodevelopmental disorders and consistent use of measures. Longitudinal designs should be employed to examine sex differences over time. Other moderators such as cognitive abilities, socio-economic status, ethnicity and comorbidities should be explored. Additionally, examining sex differences in community samples would be important in understanding whether there are variations in reported sex differences between clinical versus community samples. Lastly, other biological markers (e.g., genetics, brain) of sex differences should be evaluated.

### Implications

Understanding potential sex differences in social and communication outcomes across neurodevelopmental disorders is critical in elucidating the biology of these disorders. In addition, this study suggests that other unidentified factors including potentially IQ and population characteristics may explain the significant heterogeneity observed across the studies and should be included in future studies.

## Conclusions

The present study did not identify significant sex differences in social communication between ASD, ADHD, and controls. However, the limited number of studies, small female samples, and heterogeneity of measures/tools used, suggests that conclusions may not be drawn with confidence until larger longitudinal studies that address these issues. We argue that the overlap on the social–communication domains between the two disorders is not well characterized in the current literature and can only be resolved when participants with ASD and ADHD are recruited in single cohorts and evaluated by similar measures to understand whether there are systematic differences in the types of social–communication deficits observed or whether there are overlapping subgroups across both disorders with unique patterns of deficits.

## Data Availability Statement

The R-Script used in the present study is available upon request.

## Author Contributions

TM contributed to conceptualization, did data analysis, and is primarily responsible for manuscript preparation. AD contributed to the data analysis and manuscript. JB participated in the design, of the study, cosupervised data analytic approaches, and revised and edited manuscript. NM helped with the data analysis and revised manuscript. P-YW is the librarian on the study and assisted with the search, selection, and the process of the systematic review and has revised manuscript. AI has significantly contributed to the manuscript preparation. EA supervised all procedures in this study and manuscript. All authors have read and approved the final manuscript.

## Funding

This study was funded by the Ontario Brain Institute−Province of Ontario Neurodevelopmental Disorders (POND) Network (grant number: IDP-PND-2018) and Ontario Graduate Scholarship.

## Conflict of Interest

EA has served as a consultant to Roche, has received grant funding from Sanofi Canada and SynapDx, has received royalties from APPI and Springer, and has received kind support from AMO Pharmaceuticals.

The remaining authors declare that the research was conducted in the absence of any commercial or financial relationships that could be construed as a potential conflict of interest.
