# Supplementary material for: Meta-Analysis of Sex Differences in Social and Communication Function in Children With Autism Spectrum Disorder and Attention-Deficit/Hyperactivity Disorder
Source: Front Psychiatry. 2019 Nov 4;10:804. doi: 10.3389/fpsyt.2019.00804 (PMC6844182; doi:10.3389/fpsyt.2019.00804)
Supplement: Supplementary file 1 [file DataSheet_1.docx]

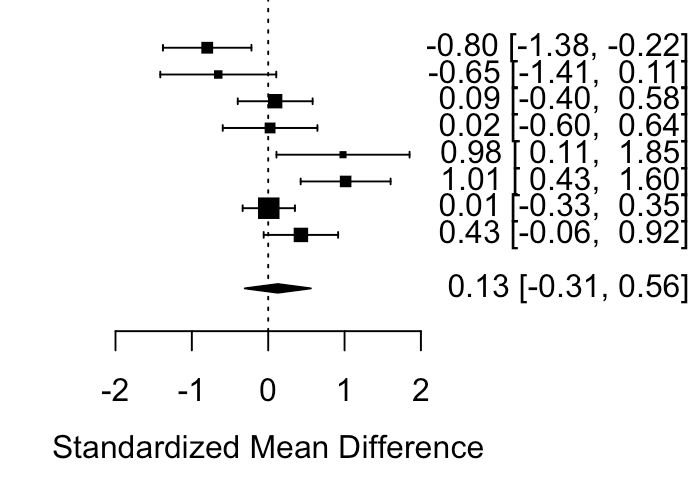


Study 1: Cholemkery, Mojica-SRS Total

Study 2: Cholemkery, Kitzerow -SRS Total

Study 3: May-SRS Total

Study 4: Solomon-SRS Total

Study 5: Sedgewick-SRS-2 Total

Study 6: Head-Friendship Questionnaire

Study 7: Horiuchi-SDQ Prosocial

Study 8: Park-ADI-R Social

Combined Effect for ASD:

**ASD Social:**

**Female-Male Difference**


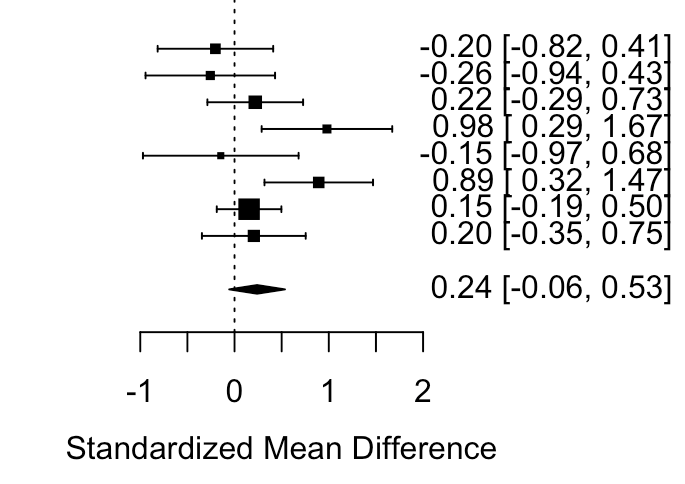


Study 1: Cholemkery, Mojica-SRS Total

Study 2: Cholemkery, Kitzerow -SRS Total

Study 3: May-SRS Total

Study 4: Solomon-SRS Total

Study 5: Sedgewick-SRS-2 Total

Study 6: Head-Friendship Questionnaire

Study 7: Horiuchi-SDQ Prosocial

Study 8: Park-ADI-R Social

Combined Effect for TD:

**TD Social:**

**Female-Male Difference**

**Supplemental Figure 1:** Forest plots of sex differences in social abilities within ASD and TD


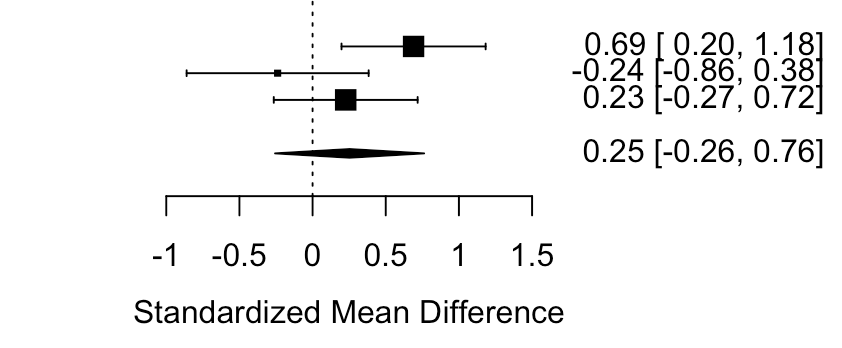


Study 1: Park-ADI-R-Nonverbal Comm

Study 2: Solomon-CCC2-General Communication Comp

Study 3: May-CCC2-General Communication Comp

Combined Effect for ASD:

**ASD Communication:**

**Female-Male Difference**


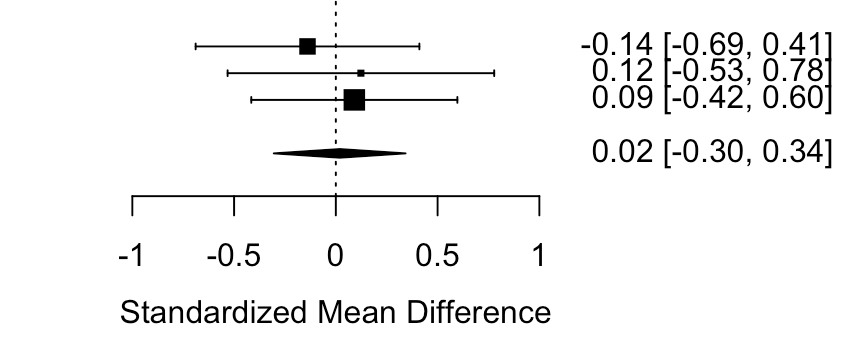


Study 1: Park-ADI-R-Nonverbal Comm

Study 2: Solomon-CCC2-General Communication Comp

Study 3: May-CCC2-General Communication Comp

Combined Effect for TD:

**TD Communication:**

**Female-Male Difference**

**Supplemental Figure 2:** Forest plots of sex differences in communication abilities within ASD and TD

**Supplemental Figure 3:** Forest plots of sex differences in social abilities within ADHD and TD


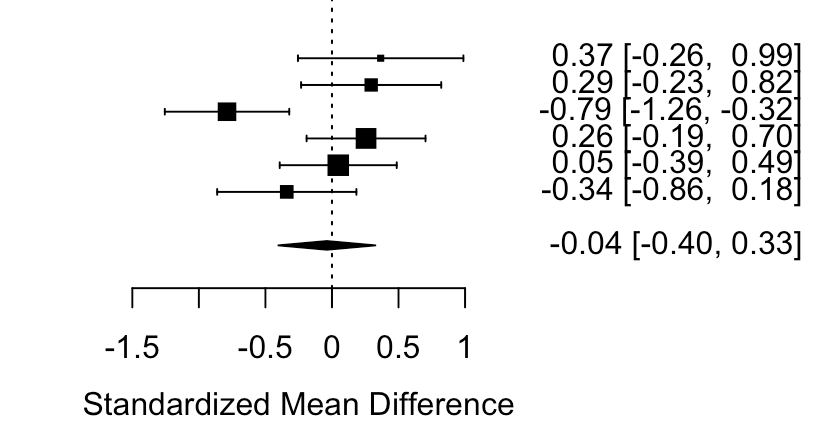


Study 1: Marton-My Child-Parent Empathy

Study 2: Mikami-Quality of Play-Conflict Scale

Study 3: Biederman-SAICA-Activity with Peers

Study 4: Graetz-CBCL-Social Problems

Study 5: Skolgi-CBCL-Social Problems

Study 6: Rucklidge-CDI-Interpersonal Problems

Combined Effect for ADHD:

**ADHD Social:**

**Female-Male Difference**


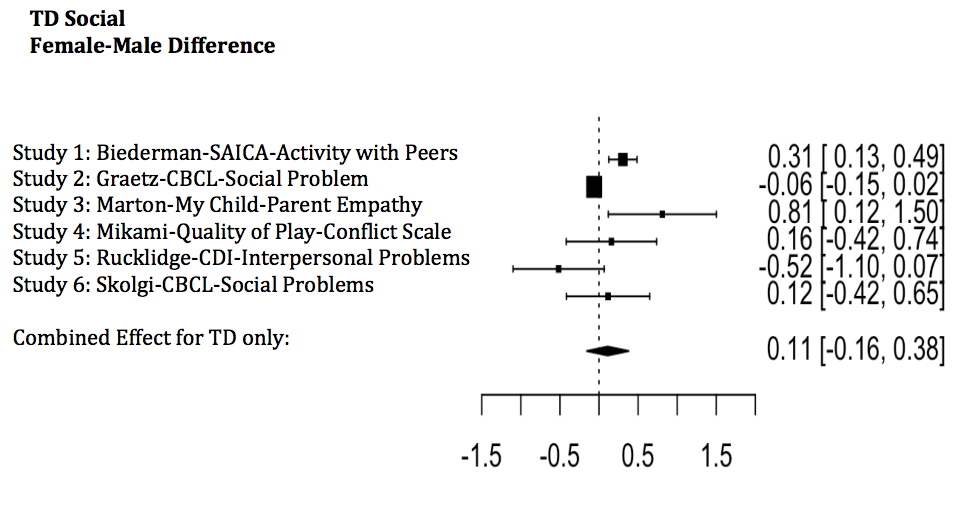


| Author (year)  Supplemental Table 1: Quality Assessment Tool for Observational Cohort and Cross-Sectional Studies for ASD Studies | 1.  Research question or objectives clearly stated | 2.  Study population clearly specified and defined | 3. Participation rate of eligible persons at least 50% | 4.  Subjects selected or recruited from the same or similar populations (including the same time period)? Inclusion and exclusion criteria prespecified | 5.  Sample size justification provided, power description, or variance and effect estimates provided? | 6.  Exposure of interest measured prior to outcome being measured | 7.  Timeframe sufficient so that one could reasonably expect to see an association between exposure and outcome if existed. | 8.  For exposures that can vary in amount or level, did the study examine different levels of the exposure as related to the outcome (e.g., categories of exposure , or exposure measured as continuous variable)? | 9.  Exposure measures (independent variables) clearly defined, valid, reliable, and implemented consistently across all study participants. | 10.  Exposure assessed more than once over time | 11.  Outcome measures (dependent variables) clearly defined, valid, reliable, and implemented consistently across all study participants? | 12.  Outcome assessors blinded to the exposure status of participants. | 13.  Loss to follow-up after baseline 20% or less. | 14.  Key potential confounding variables measured and adjusted statistically for their impact on the relationship between exposure(s) and outcomes(s)? |
| --- | --- | --- | --- | --- | --- | --- | --- | --- | --- | --- | --- | --- | --- | --- |
| Cholemkery, Mojica (2014) | Y | Y | N | N | Y | N(cross-sectional studies are a no) | N (cross-sectional studies are a no) | N | Y | N | Y | NA | N | IQ: Y  Age: Y  SES: N |
| Cholemkery, Kitzerow (2014) | Y | Y | N | N | Y | N (cross-sectional studies are a no) | N (cross-sectional studies are a no) | N | Y | N | Y | NA | N | IQ: Y  Age: Y  SES: N |
| Head et al. (2014) | Y | Y | N | N | N | N(cross-sectional studies are a no) | N (cross-sectional studies are a no) | N | Y | N | Y | NA | N | IQ: N  Age: N  SES: N |
| Horiuchi et al. (2014) | Y | Y | N | N | N | N (cross-sectional studies are a no) | N (cross-sectional studies are a no) | N | Y | N | Y | NA | N | IQ: Y  Age: Y  SES: N |
| May et al. (2016) | Y | Y | N | N | N | N(cross-sectional studies are a no) | N (cross-sectional studies are a no) | N | Y | N | Y | NA | N | IQ: Y  Age: Y  SES: N |
| Park et al. (2012) | Y | Y | N | N | N | N (cross-sectional studies are a no) | N (cross-sectional studies are a no) | N | Y | N | Y | NA | N | IQ: Y  Age: Y  SES: N |
| Sedgewick et al. (2016) | Y | Y | N | N | N | N (cross-sectional studies are a no) | N (cross-sectional studies are a no) | N | Y | N | Y | NA | N | IQ: Y  Age: Y  SES: N |
| Solomon et al. (2012) | Y | Y | N | N | N | N (cross-sectional studies are a no) | N (cross-sectional studies are a no) | N | Y | N | Y | NA | N | IQ: Y  Age: Y  SES: N |

*Y: Yes; N: No; NA: Not Applicable; IQ: Intelligence Quotient; SES: Socioeconomic status*

Supplemental Table 2: Quality Assessment Tool for Observational Cohort and Cross-Sectional Studies for ADHD Studies

| Author (year) | 1.  Research question or objectives clearly stated | 2.  Study population clearly specified and defined | 3.  Participation rate of eligible persons at least 50% | 4.  Subjects selected or recruited from the same or similar populations (including the same time period)? Inclusion and exclusion criteria prespecified | 5.  Sample size justification provided, power description, or variance and effect estimates provided? | 6.  Exposure of interest measured prior to outcome being measured | 7.  Timeframe sufficient so that one could reasonably expect to see an association between exposure and outcome if existed. | 8.  For exposures that can vary in amount or level, did the study examine different levels of the exposure as related to the outcome (e.g., categories of exposure , or exposure measured as continuous variable)? | 9.  Exposure measures (independent variables) clearly defined, valid, reliable, and implemented consistently across all study participants. | 10.  Exposure assessed more than once over time | 11.  Outcome measures (dependent variables) clearly defined, valid, reliable, and implemented consistently across all study participants? | 12.  Outcome assessors blinded to the exposure status of participants. | 13.  Loss to follow-up after baseline 20% or less. | 14.  Key potential confounding variables measured and adjusted statistically for their impact on the relationship between exposure(s) and outcomes(s)? |
| --- | --- | --- | --- | --- | --- | --- | --- | --- | --- | --- | --- | --- | --- | --- |
| Biederman et. al (2005) | Y | Y | N | Y | N | N | N | N | Y | N | Y | NA | N | IQ: Y  Age: Y  SES:Y |
| Graetz et al. (2005) | Y | Y | N | Y | N | N | N | N | Y | N | Y | NA | N | IQ: N  Age: N  SES: N |
| Marton et al. (2008) | Y | Y | N | N | N | N | N | N | Y | N | Y | NA | N | IQ: N  Age: N  SES: Y |
| Skogli et al. (2013) | Y | Y | N | N | N | N | N | N | Y | N | Y | NA | N | IQ: N  Age: N  SES: N |
| Rucklidge et al. (2001) | Y | Y | N | N | N | N | N | N | Y | N | Y | NA | N | IQ: Y  Age: Y  SES: Y |
| Mikami et al. (2011)  *Y: Yes; N: No; NA: Not Applicable; IQ: Intelligence Quotient; SES: Socioeconomic status* | Y | Y | N | N | N | N | N | N | Y | N | Y | NA | N | IQ: Y  Age: N  SES: N |
